# Epidemiologic profile and clinical course of four confirmed rickettsiosis cases in Southern Mexico during 2016

**DOI:** 10.1002/ccr3.1303

**Published:** 2017-11-29

**Authors:** Karla R. Dzul‐Rosado, Nina Mendez, Cesar Lugo‐Caballero, Jorge E. Zavala‐Castro, Salvador Gomez‐Carro

**Affiliations:** ^1^ Laboratorio de enfermedades emergentes y reemergentes Centro de Investigaciones Regionales Dr. Hideyo Noguchi Universidad Autonoma de Yucatan Merida México; ^2^ School of Medicine, Health Sciences Campus Universidad Marista de Merida Merida México; ^3^ Hospital Epidemiology Department Epidemiologic Surveillance Unit at O′Horan General Hospital Merida México

**Keywords:** Disease vector, human, Rickettsia infections, signs and symptoms, tick

## Abstract

Domestic animals can carry ticks or fleas, which constitute common vectors of rickettsial infections. The contact with them should be considered as suggestive of rickettsial infections in symptomatic patients. Misdiagnosis might occur in regions where other vector‐borne diseases are endemic. Anamnesis is essential for an accurate clinical diagnosis.

## Introduction

In Mexico, the *Rickettsia* species are causal agents of diseases such as murine typhus, Rocky Mountain, and Mediterranean spotted fevers, which cause infections with various clinical manifestations in humans 1.

*Rickettsia* spp. are transmitted to human through contact with infected arthropods. Wild and domestic animals are carriers of ectoparasites like ticks, flees, or dust mites 2, 3, 4.

In the State of Yucatan, in Southern Mexico, recent studies have confirmed diseases caused by *R. typhi* in humans. During 2015, the federal board of epidemiology notified 26 new cases of murine typhus in the State of Yucatan, situating the state as fourth place nationwide. In 2016, four cases of rickettsioses were confirmed 5, 6.

Studies from Mexico suggest that misdiagnosis and underreporting of rickettsial infections might not be uncommon, but also that poor outcomes could be associated with a late medical diagnosis. An outdated diagnosis can be potentially fatal, given that rickettsial infections require specific antimicrobial treatment for a satisfactory resolution. Thus, it is important to report the clinical and sociodemographic characteristics that could orient the diagnosis in endemic regions, in order to guide medical practice, and thus improve the prognosis of infected patients 7, 8.

The objective of this study is to present the clinical manifestations, therapeutic approach, and principal sociodemographic characteristics that were associated with four laboratory‐confirmed rickettsial infections.

We evaluated four clinical cases that were processed and confirmed by the epidemiological surveillance system through their central epidemiology laboratory. The medical history of the patients was integrated by the medical attention registry and based on the anamnesis of the patients and their family members.

## Results

### Case 1

A two‐year‐old male patient who lived in an urban coastal community. The patient had been diagnosed with dengue exclusively under clinical criteria and treated with nimesulide and acetaminophen. The patient was taken to the community's emergency pediatric service by his mother having a 39°C fever, emesis, and a generalized pruritic erythematous maculopapular rash treated. Upon his arrival to the emergency ward, he had developed generalized jaundice (skin and mucosae), hepatomegaly, splenomegaly, and lymphangitis along with lymph node enlargement in the armpit and groin regions. The only relevant antecedent mentioned by the mother was that the child was under the daily care of his grandmother, while the mother worked. The children spent weekdays with his grandmother, where they cohabitated with a small‐race dog that was kept in the backyard while the boy was present, because, according to the mother, *“*the dog continuously had ticks.”

The patient was transferred to complete his diagnosis and treatment in a general public hospital, after being diagnosed with “multiple‐drug intoxication versus viral hepatitis*.”* Blood serum values (see Table 1) of C‐reactive protein, lactic dehydrogenase, and liver function biomarkers were abnormally elevated, in addition to leukocytosis, neutrophilia, and the presence of lymphopenia. Dengue, viral hepatitis, toxoplasmosis, and myeloproliferative syndrome were discarded during his hospitalization. Immune histochemical study of the lymph nodes showed follicular hyperplasia with paracortical and sinusoidal expression, in addition to an increase in the dendritic cells were identified, which are compatible with an infectious process. Ten days after the fever first started, blood samples were drawn to discard rickettsiosis by IgM in blood serum. The patient was transferred to the intensive care unit after developing sepsis; his results were positive for Rickettsia rickettsii (1:128); therefore, treatment with doxycycline was administered, but death followed a few hours after initiating treatment due to multiple organ failure.

**Table 1:** Blood test values of the four patients with confirmed rickettsioses

Test	Case 1	Case 2	Case 3	Case 4	Units	Reference values
Hemoglobin	14.6	13.0		12.3	g/dL	10.7–13.9
Hematocrit	45.7	38.9		37.2	%	33–44
Platelets	140.0	58.0		42.0	×10^3^/UL	150–350
Leukocytes	27.2	12.1		11.4	×10^3^/UL	4.000–11.000
Neutrophils (%)	90.2	92.0		89.8	×10^3^/UL	1.4–6.5
Lymphocytes (%)	1.8	1.0		9.1	×10^3^/UL	1.2–3.4
Monocytes (%)	0.4	0.3		1.0	×10^3^/UL	0–0.4
PT	25.6			28.8	sec	11.5–15.3
PTT	87.2		62.7		sec	35.1–46.3
Glucose	181	102	45		mg/dL	75–110
Urea	57.8	6.4	115.6		mg/dL	10.00–50.00
Creatinine	0.2	0.6	2.0		mg/100 mL	0.2–0.4
BUN	27	3	54		mg/dL	6.00–18.00
Na	128	142	128		mmol/L	136–144
Cl	88	114	92		mmol/L	101–111
K	4.5	3.9	3.5		mmol/L	3.6–5.1
TB	2.8	3.4	2.6		mg/dL	1.2
UB	2.0	2.0	1.8		mg/dL	0–0.25
CB	0.8	1.4	0.8		mg/dL	0–0.75
AST	481		179		U/L	0–31
AP	205				U/L	64–306
LDH	1979	1940			U/l	225–450
Albumin	1.1				g/dL	3.8–5.1
Globulin	1.5				g/dL	2.8–3.1
Total proteins	2.6				g/dL	6.6–8.7

AST, aspartate aminotransferase; TB, total bilirubin; UB, unconjugated bilirubin; CB, conjugated bilirubin; UN, urea nitrogen; LDH, lactate dehydrogenase; AP, alkaline phosphatase; PT, prothrombin time; TTP, partial thromboplastin time.

### Case 2

A 19‐year‐old male a photographer who lived in a suburban housing unit and had no personal pathological antecedents, nor history of food or environmental allergies. The patient had no surgical or traumatic antecedents. As a precedent, he referred to living with a domestic dog that has access to the interior of the house. The dog was recently diagnosed as terminally ill by a veterinarian, after finding exterior “tumors” covered with ticks. However, the owners of the dog did not begin any treatment, nor did they restrict its access to the interior of the house and declined the option of euthanasia.

**Figure 1:**
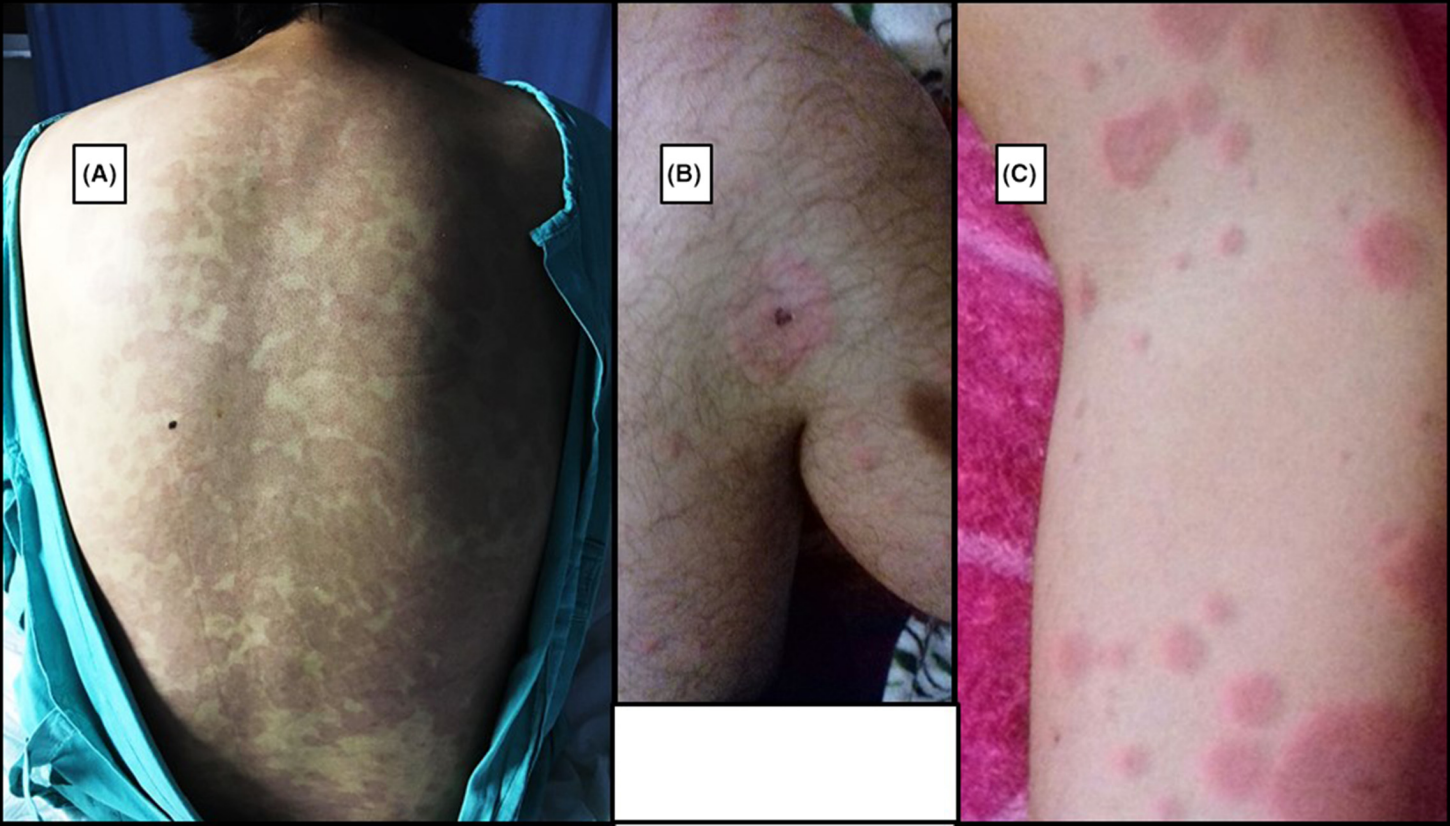
Images of patients with acute rickettsial infection. (A and B) 19‐year‐old patient erythema and target lesion. (C) 12‐year‐old patient.

The patient′s symptomatology included high fever, asthenia, adynamia, cephalalgia, and a pruritic erythematous maculopapular rash with target skin lesions on his legs, which later spread to his torso and arms. When he initially sought for medical attention, he was diagnosed with dengue and was treated with non‐steroid anti‐inflammatory drugs and topic antihistamines. The fever ceased 2 days later. One week following symptom onset, the patient presented with fever again, but this time accompanied by abdominal pain, nausea, and dizziness. On his next medical consultation, he was prescribed with two different antibiotics in addition to anti‐inflammatory and steroids. His rash was intensified (Fig. 1A and B) and accompanied by diffuse muscular pain. He was transferred to the emergency room in the nearest general hospital, where he was diagnosed with dengue and adverse drug reaction leading to hepatitis.

During his hospitalization, he was evaluated by dermatologists as a case of adverse drug reaction leading to hepatitis, for that reason, he was treated with acetaminophen exclusively. The patient developed hyperbilirubinemia (elevated indirect bilirubin), thrombocytopenia, elevated lactate dehydrogenase and high C‐reactive protein, and leukocytosis with neutrophilia (92%) and lymphopenia.

Two days later, due to the lack of clinical improvement and the exposure to ticks, polymerase chain reaction (PCR), IgM, and IgG were tested for *Rickettsia typhi* and *Rickettsia rickettsii,* with IgM‐ and PCR‐positive results for *Rickettsia rickettsii* (1:64). The patient was treated with doxycycline and improved satisfactorily.

### Case 3

Male 9‐year‐old patient with no previous pathological, traumatic, or surgical antecedents of importance. The patient lived in a small very low‐income community of <2000 inhabitants and did not cohabitate with pets, but was accustomed to playing with his neighbors’ hunting dogs outside the house (in this rural region of Mexico, it is common for dogs to walk freely in the streets that are in the proximity to their owners’ houses). The patient initially developed high fever and a pruritic erythematous maculopapular rash on the abdomen. The initial diagnosis was chikungunya. Treatment involved only acetaminophen administration during the first 6 days at a conventional dose. Two days later, the fever returned, and articular pain ensued, in addition to the onset of paresthesia and a holocranial headache that diminished with the patient in resting position; the mother consulted in the rural medical unit, and the patient received initial treatment. The patient developed dizziness and emesis (four times in a 3‐h period) and intense abdominal pain that was treated with metoclopramide and ranitidine, but no laboratory tests were indicated. On the 10th day, the patient's mother consulted over the difficulty in keeping her son awake. On this occasion, the patient was admitted to the clinic for further observation, noticing a progressive neurological deterioration; a few minutes after his admission, the patient experienced tonic–clonic seizures and was administered benzodiazepine. Subsequently, the patient was transferred to a general hospital. In the emergency room, the patient arrived dehydrated and sedated, with persistent generalized maculopapular exanthema, hepatomegaly, splenomegaly, and impaired distal circulation with coldness in both feet. The laboratory results showed severe thrombocytopenia, high titers of hepatic enzymes, as well as leukocytosis with neutrophilia (91%). The patient was transferred to pediatric intensive care after developing bilateral hemothorax, hepatic failure, and after a few hours, hypovolemic shock. Measures were taken to restore blood volume and cease internal bleeding. The patient was tested for rickettsiosis and received antibiotic treatment with imipenem. The patient developed multiple organ failure resulting in death. The IgM and IgG laboratory test results were positive for *Rickettsia spp*.

### Case 4

A healthy 12‐year‐old male of high socioeconomic status, debuted with fever accompanied by anorexia, adynamia, and a generalized maculopapular erythema. The only precedent of relevance was that the patient had resided in a rural community with his relatives for a short period of time during the holidays; those relatives had recently been under treatment with ivermectin due to lice infestation. The mother of the minor mentioned that she was part of a rescue and adoption network of abandoned dogs and cats, occasionally using her personal vehicle to transport the animals.

The patient was taken by her mother to consultation and was clinically diagnosed with chikungunya, but the diagnose was not confirmed with laboratory tests. The patient was treated with penicillin and non‐steroid anti‐inflammatory drugs to control the fever. Ten days after symptom onset, the patient continued with the erythema (Fig. 1C) and intermittent fever spikes, but the weakness and weight loss motivated the mother to seek out medical attention again. On this occasion, samples were taken to confirm chikungunya, yielding negative results. Under the “suspected diagnosis of salmonellosis,” the doctor administered antibiotic treatment with ampicillin. However, the patient's fever persisted for two more weeks, and he lost five kilograms of bodyweight. Also, he continued with sleepiness, weakness, and emesis. In one instance, the patient suffered a faint which caused him to fall from his own height while he was at school in a recess.

After almost a month since the onset of symptoms, the patient was taken to a different physician. Laboratory tests confirmed rickettsiosis, and the patient was treated with doxycycline after receiving positive results for IgM *Rickettsia typhi*. Three weeks later, during a follow‐up visit, the patient tested positive for IgG *Rickettsia typhi* (1:256) *and Rickettsia rickettsii* (1:64). Although the patient had not recovered his previous weight, the clinical evolution of the patient was satisfactory.

## Discussion

We have presented four confirmed cases of rickettsiosis with common clinical manifestations, but with different approaches, treatments, and outcomes.

In three of the four cases, the environmental origin of the infection was in an urban setting, which differs from previous reports which identify the origin in rural settings of southeastern Mexico. Congruent with previous reports of rickettsiosis, the cases presented in this study suggest that the infection originated from contact with ticks residing on domestic animals 4, 9.

Three of the cases occurred in pediatric patients, including two cases with fatal outcomes and one with a satisfactory evolution. Two non‐exclusionary hypotheses could explain these outcomes. In the first instance, the fatal cases resulted from infection by *R. rickettsii* which may be either a more aggressive species in humans or increases outcome severity when a simultaneous infection with *R. typhi* exists. Another explanation is related to the health conditions inherent to each patient; for instance, the patient in case study 4 was older, of high socioeconomic status and, therefore, probably a healthier environment than the other two pediatric patients. Thus, despite experiencing a severe infection, the patient was able to reestablish his health 10, 11, 12.

In the two fatal cases reported here, the presence of hepatic and spleen enlargement was shown to be an indicator of the severity of the infection, as well as the lymphadenopathy and the neurological affectation, given that they proceeded to deterioration and death. The presence of lymphadenopathy has previously been described as a sign of rickettsial infection, and neurological manifestations have been reported to be related to the mortality associated with rickettsial infections; however, the presence of hepatomegaly and splenomegaly had not been commonly considered as significant signs 13, 14, 15.

In each of the four cases, the medical services had poorly diagnosed the patients’ clinical manifestations of fever and rash as viral entities transmitted by the *Aedes* vector and even when the parameters of hematic biometrics did not suggest the presence of a viral infection (given the existence of neutrophilia with lymphocytosis). The doctors who made the diagnoses did not initially suspect the presence of a bacterial infection. Even though the emergent and reemerging illnesses transmitted by vectors of the *Aedes* genre have been the cause of recent epidemics in the region, it is always important to conduct a pertinent anamnesis to distinguish between different etiologies, particularly when the existing evidence does not support a viral profile. There have been reports of over diagnosis of endemic illnesses in regions of the world where dengue has caused endemic spikes, which would suggest that it is necessary to strengthen the medical knowledge of differential diagnosis in places with endemic infections, as is seen in the southeast of Mexico. This reinforces the hypothesis that orients toward an important underreporting of rickettsial illnesses, which are mainly derived from the incorrect suspect diagnosis 16, 17.

It is extremely important to promote sanitary and hygienic measures that limit the transmission of the rickettsiosis through care for the hygiene and health of companion dogs, to achieve a social conscience about the link between the well‐being of domestic animals and that of human health 18.

We observed that the presence of antecedents of contact with the arthropod vector in domestic dogs was readily advised by family members of patients. However, we cannot assure that it was intentionally investigated during the initial anamnesis in the first medical consultation. Without a doubt, the anamnesis is an orienting process and it is relevant in the process of differential diagnosis, which is why it should not be ignored during the initial medical consultations nor in the elaboration of hospital clinical histories.

## Authorship

All authors provided conceptual contributions and approved the manuscript's final version. KRD‐R: conceived, designed and developed the biomedical analysis, obtained relevant pathological antecedents of all four patients and drafted the manuscript. NM: Drafted the first version of the manuscript, conceived, designed and developed clinical anamnesis and interviews for sociodemographic and family related information. CL‐C: contributed with the biomedical analysis, obtained relevant pathological antecedents and provided information for the final manuscript. JEZ‐C: supervised the biomedical and molecular diagnosis, provided conceptual contributions for the correlation between laboratory results and clinical features. SG‐C: provided conceptual contributions, critically revised the manuscript and obtained from the patients and their families relevant epidemiological surveillance.

## Conflict of Interest

None declared.
